# An unusual cause of post-operative orbital edema in a child after general anesthesia

**DOI:** 10.4103/1658-354X.76500

**Published:** 2011

**Authors:** Joseph D. Tobias, Narasimhan Jagannathan, Amod Sawardekar, Tarun Bhalla

**Affiliations:** *Department of Anesthesiology & Pain Medicine, Nationwide Children’s Hospital, Columbus, Ohio*; 1 *Northwestern University’s Children’s Memorial Hospital, Chicago, Illinois*

**Keywords:** *Allergic reaction*, *allergy*, *anaphylaxis*, *ocular injury*, *periorbital edema*

## Abstract

We present an unusual ocular complication during the perioperative period, bilateral orbital edema in an otherwise healthy child after an outpatient surgical procedure. Ocular complications under general anesthesia remain a rare event. When periorbital edema is present, the appropriate work-up includes ruling out the potential for an allergic event by reviewing the medications administered and serum tryptase testing. Ophthalmology consultation should be considered to exclude pathology native to the eye itself. An allergist may assist in confirming a diagnosis and for allergic testing, if indicated. In our patient, the eventual diagnosis of exclusion was that of a localized reaction to the cellophane-based eye tape.

## INTRODUCTION

The induction of general anesthesia results in the loss of protective reflexes thereby placing the patient at risk for various types of ocular damage. Appropriate eye care during the perioperative process is the responsibility of the anesthesia provider. Corneal abrasions are the most common ocular complication seen during the perioperative period.[1,2] The most effective means of preventing corneal abrasion is early eye taping immediately after the induction of anesthesia. Other potential ocular complications that may occur during anesthesia include conjunctivitis, blurred vision, chemical injury, pressure from the anesthesia mask, trauma from the accidental falling of various items (stethoscope, laryngoscope, clinician’s identification badge) onto the eye of the patient, and in rare circumstances, even blindness.[1,3] We present an unusual ocular complication during the perioperative period, bilateral orbital edema in an otherwise healthy child after an outpatient hydrocelectomy procedure.

## CASE REPORT

**Figure 1:**
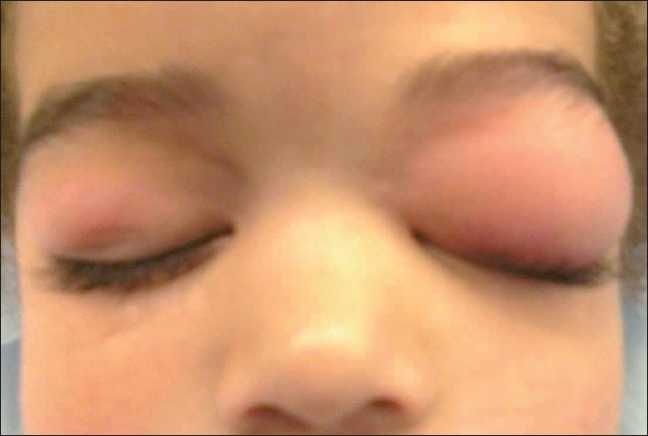
Postoperative finding of periorbital edema. The edema appears to be limited to the eyelids and is more marked in the left eye. Edema was so severe that it precluded eye opening on the left side.

Review of this patient’s hospital record and presentation of the material in this format was approved by the hospital’s Institutional Review Board. An otherwise healthy, 16 kg, 3-year-old child underwent a unilateral hydrocoelectomy under general anesthesia in the supine position. Anesthesia was induced with sevoflurane and nitrous oxide in oxygen. Eye precautions, including covering the eyes with a cellophane-based tape (Ocular Occluder; Gyrus ACMI, Southborough, MA), were performed immediately following the induction of anesthesia. The surgical procedure was started after an uneventful anesthetic induction, intravenous (IV) access, laryngeal mask airway placement, and caudal anesthesia with 0.125 % bupivacaine. The surgical procedure was completed uneventfully. Upon emergence, the eye tape was removed, and bilateral orbital edema was noted. Upon physical examination of the eyes, edema and slight erythema was noted. The edema was limited to the eyelids which was more marked in the left eye which could not be opened [Figure 1]. There was no evidence of trauma. The patient’s vital signs were stable. There was no evidence of hypotension, tachycardia, or changes in oxygen saturation. No other clinical signs of allergic phenomenon were noted such as urticaria, wheezing, or respiratory difficulty. Intravenous dexamethasone and diphenhydramine were administered. The patient was then taken to the postanesthesia care unit where he remained stable. In the subsequent hour, the orbital edema had increased in size in the left eye. An allergist was consulted to rule out an allergic phenomenon. The eye was also examined by an ophthalmologist who performed flouroscein testing to rule out a corneal abrasion. A serum tryptase levels was 4.5 ng/ml (elevated >11.5 ng/ml). The patient was admitted overnight for observation, and was discharged the next day after reduction in edema was seen in the left eyelid. The child was followed up 2 weeks after the surgical procedure and the edema was noted to have resolved.

## DISCUSSION

Although eye complications constitute 3% of all claims in the American Society of Anesthesiologists Closed Claims Database, they have a higher frequency of payment (70% versus 56%) than noneye injury claims such as medication errors and awareness during anesthesia. Several eye injuries have been reported in the literature including conjunctivitis, blurred vision, red eye, chemical injury, direct trauma, and blindness. Independent factors associated with a higher relative risk of eye injury include longer surgical procedures, greater patient age, lateral positioning during surgery, operation on the head or neck, and general anesthesia versus local anesthesia.[2] Simple measures such as early eye taping are important to prevent iatrogenic corneal abrasions. Moreover, it is suggested that clinicians not keep their stethoscope or identification badge around their necks during airway management as these items can potentially fall into the patient’s eye, causing direct injury.

In our case, we could identify no specific etiology or risk factors for the postoperative finding of periorbital edema which we initially attributed to ocular injury. There was no evidence of difficulty in mask ventilation, the surgical procedure was brief in duration, it was performed in supine position, and there was no trauma to the eyes. The differential diagnosis of orbital edema in the postoperative patient includes direct pressure from the anesthesia mask, bacterial or viral agents, trauma from the above-mentioned causes, allergy to anesthetic medications administered during the perioperative period, or a localized allergic reaction from the cellophane-based eye tape. The most common cause of allergic response in a patient undergoing anesthesia is a neuromuscular blocking agent which was not administered during this surgical procedure.[4] The potential for allergic phenomena from sevoflurane or bupivacaine is extremely low and would most likely result in more systemic manifestations such as rash, hives, bronchospasm, hypotension, or tachycardia rather that localized edema. In children, cutaneous manifestations and respiratory symptoms are present in the majority of cases of anaphylaxis.[5] Moreover, the serum tryptase level, which was normal in our patient, is generally elevated.[6] As far as other potential etiologies, consultation with ophthalmology ruled out any pathology related to the eye resulting in secondary periorbital swelling.

Based on these findings, our diagnosis of exclusion was that of a localized reaction to the cellophane-based eye tape. Silk-based tape was used to secure the intravenous cannula as well as the laryngeal mask airway. No reaction was noted in the areas where the silk tape was placed. The manufacturer of the cellophane-based eye tape was contacted and they denied any previous problems associated with the use of cellophane-based tape. In addition, they stated that the tape is manufactured using hypoallergenic materials.

In summary, ocular complications under general anesthesia remain a rare event. When present, a corneal abrasion is the most likely complication. When perioperative periorbital edema is present, the appropriate work-up includes ruling out the potential for an allergic event by reviewing the medications administered and serum tryptase testing. Moreover, risk factors associated with orbital complications in the perioperative should be considered. Opthalmology consultation should be considered to exclude pathology native to the eye itself. An allergist may assist in confirming a diagnosis and for allergic testing, if indicated. When all of the above causes are excluded, then protective cellophane eye tape used during the surgical procedure can be considered as the cause for edema.
